# Ruminant Welfare Assessment

**DOI:** 10.3390/ani15131935

**Published:** 2025-07-01

**Authors:** Marek Gaworski

**Affiliations:** Department of Production Engineering, Institute of Mechanical Engineering, Warsaw University of Life Sciences—SGGW, 02-787 Warsaw, Poland; marek_gaworski@sggw.edu.pl

Animal welfare studies are among the key areas of research contributing to the development of knowledge in the life sciences. In the Biology and Life Sciences section, it was therefore justified to propose a Special Issue on Ruminant Welfare Assessment, in order to provide a forum for the discussion of contemporary animal welfare problems. The importance of assessing the welfare of this group of animals was reflected in eight articles published in this Special Issue. Approaches to assessing the welfare of ruminants took into account many research aspects that were discussed in the individual articles.

In sustainable cattle production systems, the aim is to ensure a high level of animal welfare. In many regions, high levels of cattle welfare are achieved by providing animals with access to open spaces and grazing. This raises the problem of the effective management of the herd of animals in open spaces, which resulted in the development of the idea of a virtual fence system. The idea of virtual fences was included in two articles. In one of them, Lund et al. [Contribution 1] indicated the high costs of using individual collars for animals, which became the inspiration for studying the patterns of spatial distribution in a herd of cows, their hierarchical behavior, and the identification of cows of the highest rank; as a result of the research, premises were formulated for increasing the profitability and scalability of virtual fencing. In the second article, Staahltoft et al. [Contribution 2] investigated how effective a virtual fence system was in confining calves in a holistically managed environment where rotational grazing was implemented. The study used GPS collars to track animals and provide audible warnings and electrical impulses to keep animals within the established boundaries. The study showed that the virtual fence was able to keep calves within their designated enclosure and, over time, calves received significantly fewer electrical impulses compared to auditory warnings.

The results of the research involving the pasture-based cattle management system were presented by Romero et al. [Contribution 3]. In this research study, the protocols for assessing animal welfare in commercial conditions were developed. The feasibility of the proposed measures and methodology for assessing animal (Zebu cattle) welfare in pasture conditions was tested. It was concluded, based on the proposed methodology, that there are feasible measures to be included in protocols for the evaluation of pasture-based fattening systems under tropical conditions.

The specificity of cattle production in tropical conditions was addressed in a review article by Andrade et al. [Contribution 4]. In this study, the issue of improving cow welfare by implementing a system called compost-bedded pack barn (CBP) was developed. The application of the CBP system in open (with natural ventilation) and closed (negative-pressure ventilation system) facilities was discussed and assessed. The review focused on the following issues related to CBP: implementation, bedding, general structural and architectural features, and ambient thermal conditioning.

The livestock facility (barn) was the subject of a research study presented by Gaworski [Contribution 5]. Based on research conducted in a barn with a free-stall housing system, attention was drawn to the problem of the forced standing of cows before going to the milking parlor, taking into account an additional criterion, i.e., the level of bedding material (sand) in the lying stalls. Forcing cows to stand up can be questioned in terms of welfare. Therefore, the discussion emphasized the value of using milking robots with cows voluntarily approaching the milking stall.

Three articles in this Special Issue of Ruminant Welfare Assessment present research with small ruminants. Salari et al. [Contribution 6] investigated whether the welfare risks for dairy sheep differed according to farm size. The study considered three flock sizes, i.e., up to 500, 500–1000, and over 1000 sheep. The study results showed that on large farms, the assessment of herd management was better, but the number of daily inspections and the hygiene of water supplies were worse. Dairy sheep were also the experimental group in the study undertaken by Papakitsos et al. [Contribution 7], where the possible effects of the regrouping of dairy sheep on milk yield and milk composition, as well as behavioral indicators identifying animal welfare, were assessed. The study concluded that regrouping may have a negative impact on production and behavioral traits. It was noted that this effect was more pronounced when groups were composed of ewes of different breeds. Using small ruminants as examples, Schneidewind et al. [Contribution 8] conducted a systematic review of the knowledge on sensors developed and used in scientific research to detect rumination behavior in sheep. The literature review, including an assessment of the available sensors and their functional features, showed that none of the identified sensors were designed specifically for sheep. This highlights the need to continue designing sensor solutions that are closely adapted to the specifics of sheep. Knowledge resulting from the use of sensors has significant implications for the assessment of sheep behavior and the possibility of improving their welfare.

Research on the welfare of ruminants is not a closed topic. This research is being systematically developed, which was confirmed in the articles that make up this Special Issue. The diverse approaches to the analysis of various aspects of animal welfare indicate the wide range of possibilities for creating a forum for discussion and the exchange of scientific views on the welfare of ruminants. One forum where the presentation of research results can be continued is the Special Issue of Ruminant Welfare Assessment—Second Edition.

